# Modeling Latently Infected Cell Activation: Viral and Latent Reservoir Persistence, and Viral Blips in HIV-infected Patients on Potent Therapy

**DOI:** 10.1371/journal.pcbi.1000533

**Published:** 2009-10-16

**Authors:** Libin Rong, Alan S. Perelson

**Affiliations:** Theoretical Biology and Biophysics, Los Alamos National Laboratory, Los Alamos, New Mexico, United States of America; Emory University, United States of America

## Abstract

Although potent combination therapy is usually able to suppress plasma viral loads in HIV-1 patients to below the detection limit of conventional clinical assays, a low level of viremia frequently can be detected in plasma by more sensitive assays. Additionally, many patients experience transient episodes of viremia above the detection limit, termed viral blips, even after being on highly suppressive therapy for many years. An obstacle to viral eradication is the persistence of a latent reservoir for HIV-1 in resting memory *CD*4^+^ T cells. The mechanisms underlying low viral load persistence, slow decay of the latent reservoir, and intermittent viral blips are not fully characterized. The quantitative contributions of residual viral replication to viral and the latent reservoir persistence remain unclear. In this paper, we probe these issues by developing a mathematical model that considers latently infected cell activation in response to stochastic antigenic stimulation. We demonstrate that programmed expansion and contraction of latently infected cells upon immune activation can generate both low-level persistent viremia and intermittent viral blips. Also, a small fraction of activated T cells revert to latency, providing a potential to replenish the latent reservoir. By this means, occasional activation of latently infected cells can explain the variable decay characteristics of the latent reservoir observed in different clinical studies. Finally, we propose a phenomenological model that includes a logistic term representing homeostatic proliferation of latently infected cells. The model is simple but can robustly generate the multiphasic viral decline seen after initiation of therapy, as well as low-level persistent viremia and intermittent HIV-1 blips. Using these models, we provide a quantitative and integrated prospective into the long-term dynamics of HIV-1 and the latent reservoir in the setting of potent antiretroviral therapy.

## Author Summary

Current combination therapy can suppress viral loads in HIV-1-infected individuals to below the detection limit of standard commercial assays. However, it cannot eradicate the virus from patients. HIV-1 can generally be identified in resting memory *CD*4^+^ T cells and persists in patients on potent treatment for a long time. These latently infected cells decay slowly, but can produce new virions when activated by relevant antigens. Many patients experience transient episodes of viremia, or blips, even though they have “undetectable” plasma viral loads for many years. Here, we develop a new mathematical model describing latently infected cell activation upon random antigenic stimulation. Using the model, we show that programmed expansion and contraction of latently infected cells upon activation can generate both low viral load persistence and viral blips. Occasional replenishment of the latent reservoir may explain the different decay kinetics of the reservoir observed in clinical practice. We also show that a model with homeostatic proliferation of latently infected cells can explain persistence of low-level virus, stability of the latent reservoir, and emergence of viral blips. These results provide novel insights into the long-term virus dynamics and could have implications for the treatment of HIV-1 infection.

## Introduction

**Figure 1:**
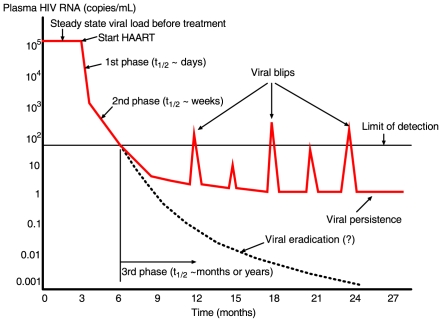
Multiphasic viral decline after potent treatment. After initiation of HAART, the plasma viral load undergoes a multiphasic decay and declines to below the detection limit (e.g., 50 RNA copies/mL) of standard assays after several months. A low level of viremia below 50 copies/mL may persist in patients for many years despite apparently effective antiretroviral treatment. Intermittent viral blips with transient HIV-1 RNA above the limit of detection are usually observed in well-suppressed patients.

Following initiation of highly active antiretroviral therapy (HAART) the plasma viral load declines with a rapid first phase, followed by a slower second phase (Figure 1, see reviews in [1]–[3]). After several months of treatment, most patients attain a level of plasma HIV-1 RNA below the detection limit (e.g., 50 copies/mL) of current standard assays [4]–[6]. However, this does not imply that viral replication has been completely suppressed by therapy. On the contrary, even in patients with “undetectable” plasma viral loads for many years, a low level of virus can be detected in plasma by supersensitive assays [7]–[9]. This phase with HIV-1 RNA below 50 copies/mL has been referred to as the third phase of viral decline after treatment [1] (Figure 1), although whether virus declines or persists at a constant level is still unresolved [9],[10]. The factors influencing this low-level viral persistence and their relative contributions have not been fully elucidated. It is possible that current HAART regimens are not completely suppressive and HIV-1 continues to replicate, particularly in some drug sanctuary sites such as the brain and testes, where drugs have poor penetration (see [11],[12] and reviews in [13],[14]). A second explanation is that HIV-1 establishes a state of latent infection in resting memory 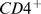 T cells [15],[16], and virus is released when these cells encounter their relevant antigens and are reactivated [17]. The latent reservoir persists in patients on HAART [18]–[20] and decays slowly, with the estimated half-life up to 44 months [21],[22]. It is more likely that both factors contribute to viral persistence. The latent reservoir releases virus that fuels ongoing viral replication, and ongoing viral replication replenishes the latent reservoir. We still lack a quantitative understanding of the relative contributions from residual ongoing viral replication and latent cell activation to the observed sustained low-level viremia.

Another line of evidence for HIV-1 persistence is the observation of transient episodes of viremia (“blips”) above the detection limit in patients on HAART (Figure 1) [7],[23]. Because viral blips are relatively rare events, their occurrence time, frequency, duration and amplitude are not well known. Di Mascio et al. [24] studied viral load time series with samples obtained approximately one month apart from 123 patients, and found that the mean blip frequency was 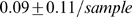, and the mean blip amplitude was 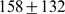 RNA copies/mL. They also suggested that a viral blip was not an isolated event but rather an extended transient episode of viremia with a duration of approximately 3 weeks [25]. In another study, Nettles et al. [26] examined the dynamics of blips with more intensive sampling over a shorter period in a cohort of 10 patients. They found that blips were brief with a mean duration of less than 3 days and had a mean amplitude of 79 copies/mL. Moreover, viral blips were not concordant on independent testing, indicating that random biological or statistical variation around a mean viral load less than 50 copies/mL might be responsible for the aberrant viral load measurements [26]. The observations by these studies may represent different phenomena, with Nettles et al. [26] observing the effects of assay variation and Di Mascio et al. [24] observing higher amplitude blips generated by occasional immune activation events [27].

The management of HIV-1 infection requires a further understanding of the mechanisms underlying low viral load persistence, stability of the latent reservoir, and occurrence of intermittent viral blips, as well as the relationships between them. We approach this through mathematical modeling. Many models, as surveyed in [28], are not capable of realistically accounting for viral load persistence since the presence of low-level replication is extremely sensitive to small changes of drug efficacy. Studies of the dynamics of the latent reservoir and viral blips are also difficult because latently infected cells are very rare [15] and blips appear to emerge randomly [24],[26]. Considering the heterogeneity of the pool of latently infected cells, a simple model was developed to study the decay characteristics of the latent reservoir [29]. Kim and Perelson [30] extended the model and showed that the latent reservoir persistence could be explained by bystander proliferation of latently infected cells. The relationship between low-level viral replication and the decay of the latent reservoir was examined in a recent study by Sedaghat et al. [31]. They developed a simple model considering the transition between latently infected and activated T cells. The results demonstrated that viral dynamics in patients under HAART might be consistent with low-level viral replication but the replication did not have much impact on the decay rate of the latent reservoir, which confirms their earlier modeling predictions [32]. Mathematical models have also been proposed to test possible mechanisms for the generation of viral blips. Jones and Perelson showed that activation of either target T cells [33] or latently infected cells [27] could result in a burst of virus production. Asymmetric division of activated latently infected cells may explain the variable decay kinetics of the latent reservoir and intermittent viral blips [34].

In this paper, we further study latently infected cell activation in response to antigenic stimulation by extending the models in [27],[30],[33]. We examine the hypothesis that stochastic activation of latently infected cells can generate intermittent viral blips and maintain low-level plasma viremia, without seriously depleting the latent reservoir in patients under HAART. The model focuses on the response of latently infected cells when they encounter their relevant antigens. We show that programmed expansion and contraction of latently infected cells can generate intermittent viral blips with realistic amplitude and duration. During the latent T cell response, part of the resultant activated T cell population reverts back to a resting state, providing a mechanism to replenish the latent reservoir. An interesting result of our model is that different potentials of activated T cells to proliferate during the response or different duration or frequency of antigenic stimulation can explain the differences between the divergent estimates of the half-life of the latent reservoir decay in HAART-treated patients [21]–[23], [35]–[38]. Using this model, we study the influence of ongoing viral replication on both the decay of the latent reservoir and persistence of low-level viremia. We perform sensitivity tests on a number of model parameters. Finally, we develop a phenomenological model that postulates density-dependent homeostatic proliferation of resting memory 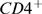 T cells. A recent experimental study supports the idea that homeostatic proliferation of latently infected cells may ensure the latent reservoir persistence without any demonstrable evidence for viral production [39]. The model can robustly describe the multiphasic viral decline following initiation of potent antiretroviral treatment. The different self-renewal potentials of latently infected cells are also able to reconcile the variable decay kinetics of the latent reservoir. Our models provide a new perspective into the possible mechanisms for viral and the latent reservoir persistence and emergence of intermittent viral blips.

## Methods

### A basic model of latent cell activation

A basic model of latent cell activation was initially developed to examine the cell populations contributing to the second-phase viral decline after administration of both reverse transcriptase (RT) and protease inhibitors [4]. Using an overall drug efficacy, 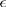, the basic model can be reduced to the simpler form [34]:
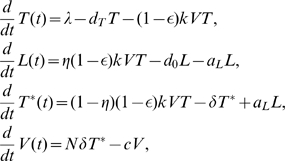
(1)where 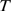 represents 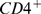 T cells that are susceptible to HIV-1 infection, 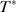 represents productively infected cells that can produce virus particles, 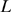 represents latently infected cells that cannot produce virus but are ready to do so once they are activated by their recall antigens, and 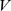 represents the total viral load. 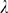 is the recruitment rate of susceptible T cells and 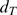 is their mortality rate. The constant 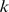 is the infection rate. 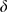 and 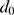 are the death rate of productively and latently infected cells, respectively. 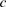 is the clearance rate of free virus. 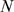 is the burst size, the total number of virions produced by an infected cell during its life span. 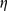 is the fraction of infections that lead to latency. 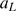 is the transition rate at which latently infected cells become productively infected cells. 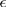 is the total drug efficacy, which is defined as 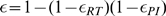 where 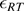 and 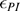 are the drug efficacy of RT and protease inhibitor, respectively.

There is only one positive steady state viral load of Eq. (1):
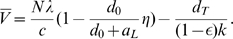
(2)It is biologically plausible if and only if 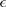 is less than a “critical efficacy”, given by
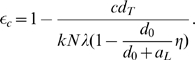
(3)If 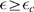, then the only steady state is the uninfected steady state, with 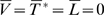, 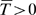. The steady state viral load (2) can theoretically achieve any positive value close to zero. However, it remains very sensitive to small changes of drug efficacy, particularly when 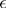 approaches 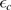 [28],[34]. Therefore, the basic model and its various variations [28] are not realistic to describe the persistence of low-level viremia in patients on HAART. Furthermore, the model cannot maintain the latent reservoir size unless the death rate of latently infected cells 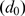 and the transition rate 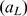 are both chosen to be very small [31]. If transient episodes of viremia also come from activation of latently infected cells as suggested in [40], then the latent reservoir will be depleted more quickly than observed in clinical studies.

### Model with programmed expansion and contraction of latently infected cells upon activation

**Figure 2:**
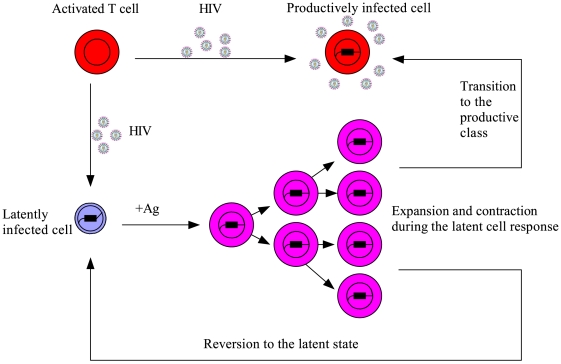
Schematic representation of the model with latently infected cell activation (Eq. (4)). Following encounter with cell-specific antigens, latently infected cells are activated and undergo programmed clonal expansion and contraction. A number of activated latently infected cells transition to the productive class and produce virions, whereas another small fraction of activated cells revert back to the latent state, providing a mechanism to replenish the latent reservoir.

Both 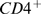 and 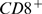 T cell responses to infectious agents (for example, lymphocytic choriomeningitis virus (LCMV) [41] and Listeria monocytogenes [42]) can be broken down into three distinct phases: expansion/activation, contraction/death and maintenance/memory [43]. Upon initial exposure to antigen, specific T cells undergo considerable antigen-driven expansion and differentiation into effector cells, whose major function is to kill infected cells. A contraction or death phase then ensues, in which the majority of activated T cells die quickly by apoptosis or activation-induced cell death. The third phase is characterized by a stable [44] or slowly decaying pool [45] of memory cells, which are formed during the response and are maintained for long periods of time. By developing mathematical models considering these phases, De Boer et al. studied the dynamics of the 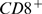 T cell response to LCMV [44] and compared them with the 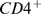 T cell response to LCMV [45]. Fitting models to experimental data, they obtained the T cell doubling time during the expansion phase and the T cell half-life during the contraction phase. These results suggest that the 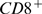 T cell response has faster kinetics in almost every aspect than 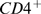 T cells [45]. Jones and Perelson [33] developed a model that accounts for both HIV infection and the programmed cascade of divisions during the expansion of the 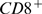 T cell response to a concurrent opportunistic infection. Using the model, they showed that target cell activation [33] or latent cell activation [27] caused by opportunistic infections was able to explain the transient low-level viremia observed in well-suppressed patients on potent treatment. Here, we reexamine the model in [27] and develop a new one in which latently infected cells are hypothesized to experience programmed expansion and contraction in response to their specific antigens, and in which a small portion of activated cells revert back to the resting state by the process that normally generates memory 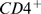 T cells (Figure 2). We investigate whether repeated latent cell activation through this type of programmed response can generate intermittent viral blips with reasonable amplitude and duration, and whether the replenishment of latently infected cells can control the decay of the latent reservoir.

Let 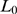 represent the concentration of resting latently infected 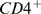 T cells. These cells on encounter with their relevant antigens may enter the class of activated cells, 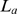. One model describing the programmed expansion and contraction of latently infected cells upon antigenic stimulation is as follows:
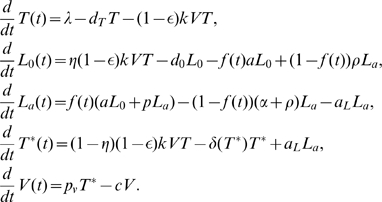
(4)When the antigen is present, resting latently infected cells, 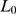, are activated into the activated class 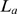 with rate constant 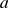. The function 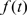 determines the times at which antigen is present at concentration sufficiently high to activate cells. Activated cells proliferate at rate 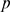. Once the antigen concentration falls, we assume there is a contraction phase, in which activated cells die or apoptose at rate 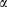, or revert to the resting state at rate 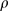. In addition, activated latently infected cells transition into productively infected cells at rate 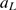 during the entire response.

As suggested by [28],[46], we use a density-dependent death rate of productively infected cells in order to reduce the sensitivity of the steady state viral load to changes of drug efficacy. The biological justification for the density-dependent cell death rate is as follows: productively infected cell can be killed at a rate that depends on the density of effector cells. The population size of effector cells can be further assumed to be proportional to the density of infected cells. Thus, the death rate of productively infected cells can be assumed to be a function of the infected cell density. We choose a simple power-law function, 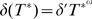, as used in [28],[46], where 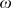 controls the size of the immune effect on the death rate. Holte et al. [46] obtained estimates of 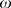 by fitting the density-dependent decay model to patient data. Because the model includes a density-dependent infected cell death rate, we have to decouple the viral production rate from the cell death rate. We assume virus is produced at a constant rate, 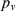, per productively infected cell, 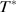. For simplicity, we assume 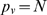. A modification of this model will be given later to study viral persistence without the assumption of density-dependent infected cell death.

We employ a basic “on-off” model, which has previously been used to describe the 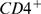 and 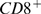 T cell responses to viral infection [44],[45], to approximate the antigenic stimulation of latently infected cells instead of explicitly modeling the interaction between naive T cells and their specific antigens as was done in [27],[33]. The activation function, 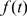, is antigen-dependent and takes on only two values: 0 if there is no activation, and 1 if there is full activation. If 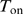 is the time at which the stimulation switches “on” and 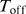 is the time at which the stimulation is “off”, then 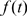 assumes the following expression:
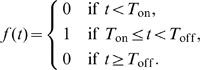
(5)We denote by 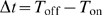 the duration that each activation lasts.

Although 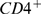 and 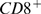 T cells both commit to clonal expansion after antigenic stimulation, 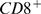 T cells typically have a higher proliferative potential both *in vitro* and *in vivo* compared with 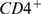 T cells [41],[47]. It has been estimated that 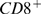 T cells divide about 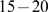 times during an acute infection with LCMV, while 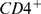 T cells divide approximately 9 times [41]. Choosing the proliferation rate of 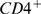 T cells as in [45], 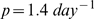, 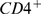 T cells can divide 8 to 12 times if the expansion phase lasts 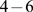 days. If 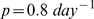, then 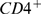 T cells only divide 5 to 7 times over the same period. With various proliferation rates, we will show that the number of times that activated latently infected cells divide upon stimulation is an influential factor that not only controls the decay of the latent reservoir but that also affects the amplitude of viral blips. Because only a small fraction of latently infected cells are specific for any given antigen, we choose 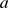 to be 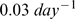. We will perform sensitivity tests on a few parameters including 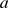. The death rate of activated cells during the contraction phase, 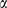, is not well-known. In [45], about 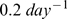 was estimated for this parameter. Because the cells activated from latently infected cells are usually not observed, here we choose a larger death rate, 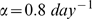, such that activated latently infected cells will decline to low levels after a relatively short period. We will discuss the effect of a smaller 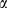 later. We also assume that a small fraction of activated cells revert back to the resting state, with rate 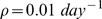 [27], whereas another portion of them transition into the productive stage with rate 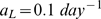. We will test our model predictions with different values of 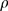 and 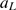.

We choose the overall drug efficacy 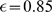 as the baseline value so that viral load can be suppressed to below the detection limit after several months of treatment. In fact, as we will show below, specific values of the drug efficacy do not strongly impact viral and the latent reservoir persistence once it exceeds a threshold called the critical efficacy. The dynamics of viral load, the latent reservoir and viral blips will also be compared with different drug efficacies. Similar arguments can be applied to the choice of the value of 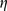, the fraction of infections resulting in latency. As long as it represents a small fraction of infections, the value of 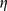 has only a minor effect. Here we choose 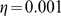 [27] as an example. The form of the activation function 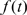 will be further discussed below. The viral burst size, 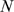, can affect the amplitude of blips generated from activation of latently infected cells. Here we use 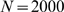 [48], although recently higher values of 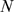 have been estimated for SIV [49]. It is not known if these higher burst sizes apply to HIV. However, if higher values of 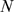 are used, then other parameters in Table 1 need to be adjusted, such as the viral clearance rate, which recent work suggests may be higher in tissue than has been estimated in blood (De Boer R., Ribeiro R. and Perelson AS, unpublished results). The other parameter values are chosen based on previously published reports and are summarized in Table 1.

**Table 1:** Variables, parameters and values used in models and simulations.

Variable/Parameter	Value	Description	Reference
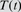	-	Target T cells	-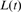	-	Latently infected cells	-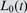	-	Resting latently infected cells	-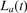	-	Activated latently infected cells	-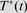	-	Productively infected cells	-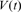	-	Viral load	-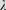	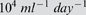	Recruitment rate of susceptible cells	[28]
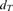	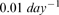	Death rate of susceptible cells	[90]
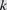	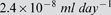	Infection rate	[50]
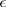	0.85	Overall drug efficacy	see text
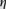	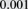	Fraction resulting in latency	[27]
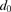	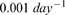	Death rate of latently infected cells	[28]
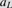	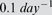	Rate of transition from latently to	see text
		productively infected cells	
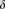	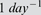	Death rate of productively infected cells	[91]
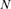	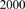	Burst size	[48]
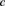	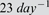	Clearance rate of free virus	[92]
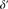	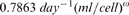	Density-dependent mortality	[28]
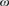	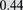	Power in density-dependent mortality function	[46]
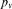	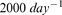	Viral production rate	[48]
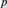	varied	Proliferation rate of activated cells	see text
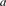	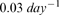	Activation rate of latent cells	see text
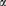	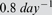	Death rate of activated cells	see text
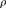	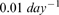	Reversion rate to latency	[27]
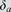	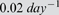	Base death rate of activated cells	[45]
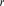	varied	Maximum proliferation rate of latent cells	see text
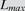	varied	Carrying capacity density of latent cells	see text
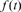	see text	Expansion function	-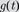	see text	Rapid contraction function	-

Since we are interested in the dynamics of the third-phase viral decline during treatment, we choose the initial viral load to be 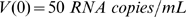. With an assumption of quasi-steady state between virions and productively infected cells, we obtain the initial condition for productively infected cells, 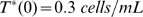. We set 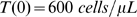 as the initial condition for target T cells [30]. The total number of latently infected cells with replication-competent viral genomes is assumed to be 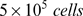 [15], 98% of which are in the lymphoid tissue and the rest are in the blood. Assuming the blood volume is 5 L, the concentration of latently infected cells with replication-competent provirus is 2 cells/mL, i.e., 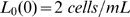. We assume there are no activated latently infected cells initially, i.e., 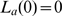.

### Model with a biphasic contraction phase

Homann et al. [41] suggested a multiphasic contraction phase in the 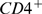 T cell response to acute LCMV infection. De Boer et al. [45] developed a mathematical model that fits the Homann data using two distinct phases of activated cell death after the peak of the response. Here, we modify model (4) by adopting a biphasic contraction phase in the latently infected 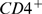 T cell response. More motivations will be addressed in the Results section after we present the results of model (4). In the modified model, the 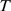 and 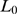 equations remain the same, while the other equations change to
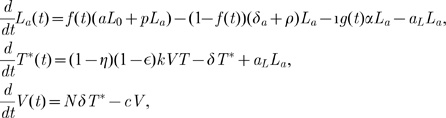
(6)where 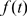 is the expansion function defined by Eq. (5). Following the expansion phase, there is a two-phase contraction: a rapid contraction phase of length 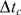, where activated cells die rapidly by apoptosis or activation-induced cell death, at a rate 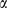, and a slower phase where activated cells die at their base mortality rate 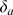. For simplicity, we assume the rapid contraction phase has the same length as the expansion phase (i.e., 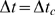). 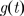 represents the contraction function. During the rapid contraction phase, 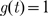, otherwise, 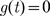.

### A phenomenological model with homeostasis of latently infected cells

A recent experimental study by Chomont et al. [39] shows that the HIV-1 latent reservoir size may be maintained by homeostatic proliferation of latently infected cells. Thus, we incorporate a logistic term representing homeostatic proliferation of latently infected cells into the basic model (1). The 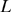 equation becomes
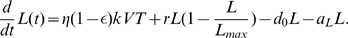
(7)The other equations for 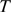, 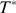 and 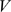 are the same as those in model (1). In Eq. (7), 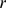 represents the maximum proliferation rate and 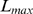 represents a threshold latent cell density, beyond which proliferation shuts off. Whether there is such a strict maximum is unclear and thus other forms of density-dependent proliferation could also be explored, such as 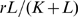, where 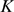 is a constant.

We choose a small base value for the transition rate, 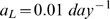 [27], because only a small fraction of latently infected cells are specific for any given antigen. We will increase the value of 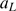 when we study latently infected cells encountering their specific antigens, which is used to model emergence of viral blips during treatment.

In order to maintain the latent cell pool during potent drug therapy, we choose the proliferation rate 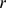 to be greater than 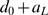, i.e., 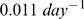. In fact, it can be proved that in the case of 100% drug effectiveness, the infected steady state exists and is locally asymptotically stable if and only if 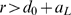.

The carrying capacity (i.e., the maximum sustainable population) of latently infected cells during therapy is unknown. Assuming 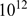 total body lymphocytes, Chun et al. [15] reported a total body load of resting 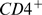 T cells with integrated HIV-1 DNA of 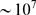 cells during the asymptomatic phase of infection. Here we allow the total body carrying capacity of latently infected cells to vary from 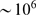 cells to 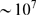 cells but then convert these numbers to a cell density in blood so as to be in the same units as the target cells, 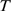. For example, if there are maximally 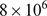 latently infected cells per patient under HAART, then the maximum density of latently infected cells in blood is 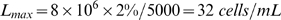 since 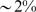 of 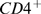 T cells are in blood and the typical 70 kg individual has about 5L of blood. We will discuss the effects of different values of 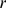 and 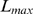 on the final model predictions.

The simulation with an initial T cell count 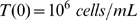 [50] and an arbitrary initial viral load value 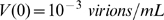 yields a set of steady state values in the absence of drug treatment: 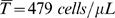, 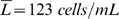, 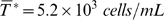 and 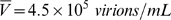. These values are set as the initial conditions when performing simulations of the model during HAART.

## Results

### Intermittent viral blips and decay of the latent reservoir

**Figure 3:**
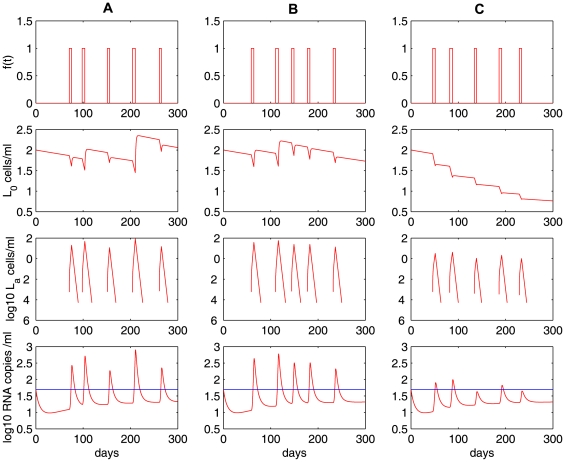
Stochastic simulations of the model with programmed expansion and contraction (Eq. (4)). The model with programmed expansion and contraction of latently infected cells can generate viral blips with reasonable amplitude and duration. 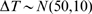, 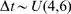. Column **A:** 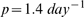. Activated latently infected cells divide about 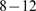 times over an interval [4],[6] days. No statistically significant decay of the latent reservoir is observed. Column **B:** 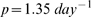. The latent reservoir decays at a very slow rate. This realization shows a half-life of 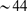 months. Column **C:** 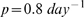. Activated cells divide about 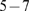 times over the same time interval. The latent reservoir decays more quickly than it does in **B**, corresponding to a half-life of roughly 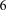 months. The other parameter values used are listed in Table 1. The blue horizontal line represents the detection limit of 50 RNA copies/mL.

Numerical simulations of model (4) show that programmed expansion and contraction of latently infected cells upon occasional antigenic stimulation can robustly generate intermittent viral blips with reasonable amplitude and duration, without seriously depleting the latent reservoir (Figure 3). We assume that latently infected cells encounter their specific antigens randomly. As an example, we assume the interval between two adjacent activations, 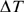, obeys a normal distribution with a mean of 50 days and a standard deviation of 10 days. If we use a Poisson process to model the encounter between latently infected cells and antigen, then we get a similar pattern of viral blips and the latent reservoir decay when the average waiting time between two encounters is assumed to be 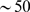 days. The duration of activated T cell proliferation during the latent cell response remains unknown. In fact, the mechanisms that control the rate and extent of T cell differentiation are complicated [47]. It may involve the amount of antigen and other types of cytokines that are present *in vivo*, the duration of antigen exposure, as well as whether T cell proliferation continues in the absence of further antigenic stimulation [51]–[54]. In our simulation, we assume the duration of activated T cell proliferation, 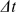, obeys a uniform distribution over the interval of 4 to 6 days such that 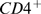 T cells divide approximately 5 to 12 times with appropriate proliferation rates (see below) [41]. A transient episode of viremia is observed every time an activation occurs. Thus, the timing and frequency of viral blips are determined by when and how often latently infected cells encounter their recall antigens. The duration of transient viremia is determined by how long the antigen is present (Figure 3). Another important characteristic of viral blips, the amplitude, ranges from 50 RNA copies/mL to roughly 500 copies/mL in our simulations. This is consistent with observations in clinical trials [24],[26],[55].

An interesting result is that the amplitude of viral blips is inversely correlated with the decay of the latent reservoir. Based on model (4), viral blips originate from activation of latently infected cells into the productive class. It was initially thought that this activation would deplete the latent reservoir quickly in HAART-treated patients because *de novo* infection of susceptible T cells is maximally inhibited by potent antiretroviral drugs and productively infected cells have a fast turnover rate. However, if the activation induces a substantial proliferation of activated latently infected cells, it can simultaneously reseed the latent reservoir as a small fraction of activated cells revert to the resting state in the formation of memory T cells. To what extent the activation replenishes the latent cell pool depends heavily on the proliferative potential of activated cells, i.e., how many daughter cells are derived from the activation of latently infected cells. In Figure 3**A**, the proliferation rate of activated cells is chosen to be 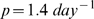, which implies that cell divisions occur 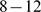 times over an interval of 4 to 6 days. In this case, the activation induces a high level of activated T cells. As a consequence, a large number of productively infected cells are generated. Thus, the amplitude of viral blips remains relatively high, and the latent reservoir is largely replenished since more activated latently infected cells revert back to the resting state. In our simulation, we did not observe a statistically significant decay of the latent reservoir (we performed the simulation over 3 years, but only plotted the first 300 days in Figure 3**A**), suggesting that the viral reservoir can be extremely stable even with effective treatment for years. This may explain the remarkable stability of the latent reservoir in some patients on HAART [21]. In Figure 3**B**, we show an example with a slightly smaller proliferation rate, 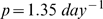. In this situation, although occasional activation can replenish the latent reservoir, the size of the latent cell pool diminishes gradually. However, the decay is very slow, with a half-life of approximately 44 months, which is consistent with some estimates [21],[22]. In Figure 3**C**, we choose the proliferation rate to be 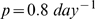, so that activated T cells divide 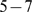 times over an interval of 4 to 6 days. In this case, a lower level of activated cells are produced, resulting in lower amplitude viral blips. The latent reservoir is depleted relatively quickly because cell activation consumes latently infected cells and the replenishment of the reservoir from activated cells is minor. Figure 3**C** shows a realization of model (4) in which the decay half-life of the latent reservoir is about 6 months, which is in agreement with the estimates in some clinical studies [23],[38].

We ran stochastic simulations of the model 30 times, recorded the number and amplitudes of viral blips, and calculated the half-life of the decay of the latent reservoir based on the change in the latent reservoir size in 300 days. The summary statistics on our simulations is given in Table 2. As the proliferation rate of activated cells 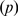 decreases, we observe that both the frequency and the average amplitude of viral blips decrease. With a smaller 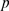, the latent reservoir size undergoes a larger decrease, corresponding to a shorter half-life of the reservoir decay. Thus, we expect an inverse relationship between the decay of the latent reservoir and the frequency (or amplitude) of viral blips. This is consistent with the experimental observations in Ramratnam et al. [23].

**Table 2:** Summary of stochastic simulations of the model, Eq. (4), with programmed expansion and contraction of latently infected cells.

Parameter value	Ave number of blips over [0, 300] days	Min blip amplitude (copies/mL)	Max blip amplitude (copies/mL)	Ave blip amplitude (copies/mL)	Change in the latent reservoir size over 300 days	Half-life of the latent reservoir decay (months)
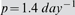	5	186 [140, 362]	693 [541, 877]	394 [298, 522]	−0.5% [−19%, +26%]	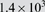 [33, -]
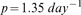	5	168 [113, 308]	524 [346, 680]	334 [263, 446]	−14% [−32%, +5.5%]	46 [18, -]
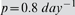	3.7 [2, 5]	61 [50, 94]	93 [71, 111]	74 [63, 98]	−65% [−67%, −62%]	6.6 [6.3, 7.3]

Abbreviations: ave (average), min (minimum), max (maximum). Values above brackets are the average values over 30 simulation runs. Values in brackets are the ranges. There are 5 antigenic activations within 300 days. When 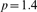 or 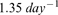, viral blip 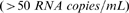 emerges each time activation occurs. When 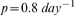, not every activation generates a viral blip. In some simulations with 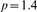 or 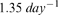, the latent reservoir size is predicted to increase and hence has no half-life.

**Figure 4:**
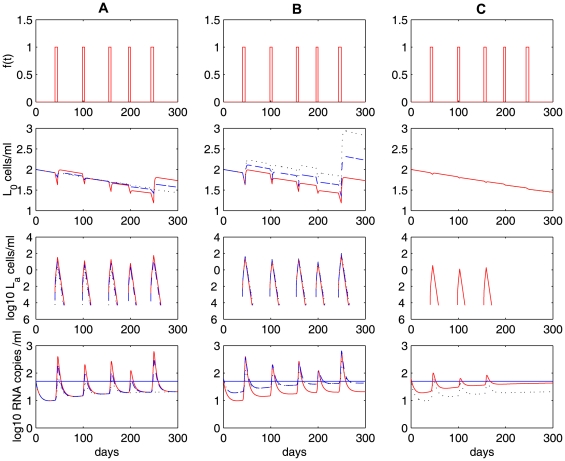
Sensitivity tests on the activation rate 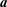 and the transition rate 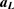 in Eq. (4). The proliferation rate of activated cells, 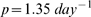, is fixed. Column **A:** the transition rate 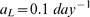 is fixed and the activation rate 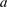 varies: 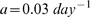 (red solid), 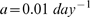 (blue dashed) and 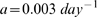 (black dotted). 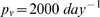 is fixed. Column **B:** the activation rate 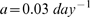 is fixed and the transition rate varies: 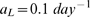 (red solid), 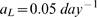 (blue dashed) and 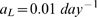 (black dotted). 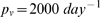 is fixed. Column **C:** 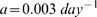 and 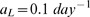 are fixed. The viral production rate varies: 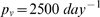 (red solid) and 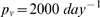 (black dotted). The other parameter values used are the same as those in Figure 3. The blue horizontal line represents the detection limit of 50 RNA copies/mL.

The fraction of resting latently infected cells that are activated by antigenic stimulation remains largely unknown. Due to the heterogeneity of latently infected cells with respect to the antigens they respond to, it is likely that a very small fraction of latently infected cells are activated by a particular antigen. We tested model predictions (Eq. (4)) with different activation rates 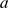. The proliferation rate of activated cells, 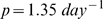, is fixed. With 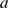 decreasing from 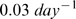 (red solid) to 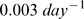 (black dotted) in Figure 4**A**, fewer activated latently infected cells are generated, which results in a more rapid decay of the latent reservoir and lower viral loads. In fact, when 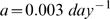, 5 activations only generate 3 viral rebounds, in which only 2 rebounds can be regarded as viral blips 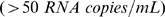. With a smaller 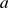 (e.g., 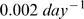), antigenic activation cannot generate viral blips with the parameter values we used. We also tested the sensitivity of generating blips to the transition rate 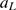 (Figure 4**B**). When 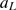 decreases from 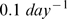 (red solid) to 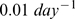 (black dotted), the viral load does not change significantly. However, a smaller transition rate leads to more substantial replenishment of the latent reservoir. Viral load also depends on the viral production rate 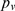. For example, when 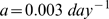, only 2 viral blips are observed with 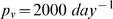 (black dotted in Figure 4**C**). If we increase the viral production rate to 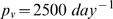 (red solid), then all the 3 rebounds are greater than 50 RNA copies/mL and thus generate observable viral blips.

**Figure 5:**
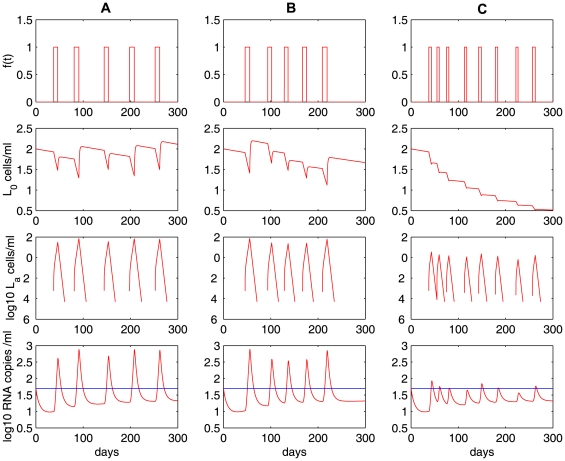
Numerical simulations of Eq. (4) with different duration and frequency of activation. We fixed the proliferation rate of activated cells to be 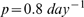. Column **A:** 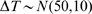, 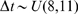. No statistically significant decay of the latent reservoir is observed. Column **B:** 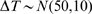, 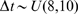. The latent reservoir decays at a very slow rate. Column **C:** 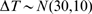, 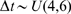. In this realization, there are 8 activations in 300 days. The latent reservoir decays more quickly than in Figure 3C. The other parameter values used are the same as those in Figure 3. The blue horizontal line represents the detection limit of 50 RNA copies/mL.

In addition to changing the proliferation rate 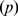 during expansion, it would also be interesting to study the effects of varying the duration 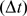 and frequency (determined by 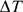) of antigenic stimulation. As an example, we showed in Figures 5**A** and 5**B** the latent reservoir decay and viral blips with different distributions of 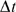. Specifically, we assumed 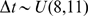 in Figure 5**A** and 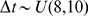 in Figure 5**B**. We fixed 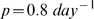 and 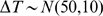 as used in Figure 3**C**. No statistically significant decay of the latent reservoir is observed in Figure 5**A**, while the latent resevoir decays at a very slow rate (with a half-life of approximately 44 months) in Figure 5**B**. This is not surprising since shorter duration of activation results in generation of less activated latently infected cells, and thus less replenishment of the latent reservoir. In Figure 5**C**, we assumed 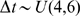 as in Figure 3**C**, but increased the frequency of activation by assuming 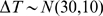. In this realization, there are 8 activations in 300 days, more than the 5 activations in Figure 3**C**. We observe that the latent reservoir decays more quickly than in Figure 3**C**. In fact, for a large proliferation rate of activated cells (e.g., 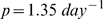 in Figure 3**B**), increasing the frequency of activation will replenish the latent reservoir more frequently and thus decrease the decay rate of the latent reservoir, whereas for a small proliferation rate (e.g., 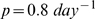) and short duration of activation (e.g., 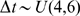), increasing the frequency of activation will accelerate the latent reservoir decay (see Figures 3**C** and 5**C**).

In summary, occasional activation of latently infected cells upon stochastic antigen encounter is able to produce a large quantity of activated T cells temporarily, and thereby generate intermittent viral blips. The blip amplitude/frequency is inversely correlated with the decay of the latent reservoir. Using different potentials of activated T cells to divide during the initial clonal expansion phase or different duration or frequency of antigenic stimulation enables us to generate the different decay characteristics of the latent reservoir observed in different clinical studies [21]–[23], [35]–[38].

### Low-level viral persistence

We have assumed a density-dependent mortality rate for productively infected cells in the model given by Eq. (4) in order to maintain a low steady state viral load when antigen is absent. The reason that viral loads decrease very quickly in the absence of activation in this model is that activated cells decline quickly to an extremely low level during the contraction phase, with not enough cells entering the productive stage. Even when we choose a smaller death rate of activated cells, for example, 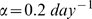 [45], activated cells still quickly decline to a very low level. If activated cells can be maintained at a low level rather than decreasing to zero quickly during the contraction phase, then low steady state viral load persistence is possible without assuming density-dependent infected cell death. A study by Chun et al. [56] revealed that a high level of HIV-1 proviral DNA persists in the activated 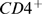 T cell compartment in infected individuals on effective antiretroviral therapy with no detectable viremia in plasma for extended periods of time. Although some of the proviruses might be defective, spontaneous release of virus was detected without any activating stimuli during overnight culture [56]. This observation argues for the persistence of infectious virus in activated 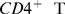 cells in patients under effective treatment. Here we modify model (4) (i.e., remove the assumption of density-dependent infected cell death and adopt a biphasic contraction phase, see Eq. (6) in Methods) and examine whether viral and the latent reservoir persistence, as well as intermittent viral blips, can be generated solely by occasional activation of latently infected cells upon encounter with relevant antigen.

**Figure 6:**
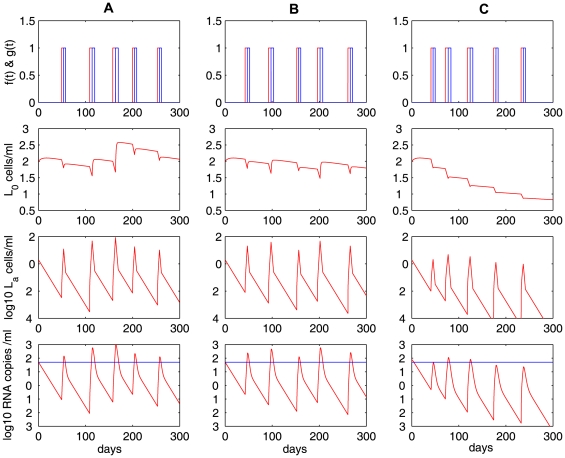
Simulations of the model with a biphasic contraction phase (Eq. (6)). The model is able to generate viral blips as well as low-level persistent viremia. The low-level viral load is maintained by a low level of activated latently infected cells during the second slower contraction phase in the latent cell response. In the first row, 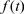 is the expansion function (red) and 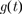 is the rapid contraction function (blue). Different proliferation rates, i.e., 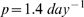 (Column **A**), 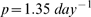 (Column **B**), and 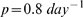 (Column **C**), result in differential decay characteristics of the latent reservoir as in Figure 3. The other parameter values used are listed in Table 1. The blue horizontal line represents the detection limit of 50 RNA copies/mL.

With 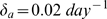 [45], 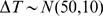, 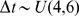, we perform numerical simulations of the model with a biphasic contraction phase. As before, we choose different proliferation rates, i.e., (**A**) 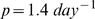, (**B**) 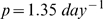, (**C**) 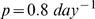, to characterize different potentials of activated cells to proliferate during the phase of expansion. Similar to Figure 3, the simulation results shown in Figure 6 exhibit three distinct decay profiles of the latent reservoir: (**A**) there is almost no decay; (**B**) the latent reservoir decays at a very slow rate; (**C**) the reservoir decays at a faster rate. The decay of the latent reservoir is inversely correlated with the amplitude or frequency of viral blips. The viral load does not decline to an unreasonably low level in the absence of antigenic stimulation. This low-level viremia is primarily maintained by a small number of activated cells that transition into the productive class during the second slower contraction phase. However, the absence of antigenic stimulation over a long time (more than 4 months in our simulation, figure not shown) will deplete activated cells, and the viral load will decrease to an extremely low level (below 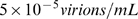, a level that can be interpreted as viral extinction [28]). Therefore, in order to obtain a low level of viremia solely maintained by latently infected cell activation, there cannot exist a very long period in which no antigenic stimulation occurs.

### The contribution of ongoing viral replication

**Figure 7:**
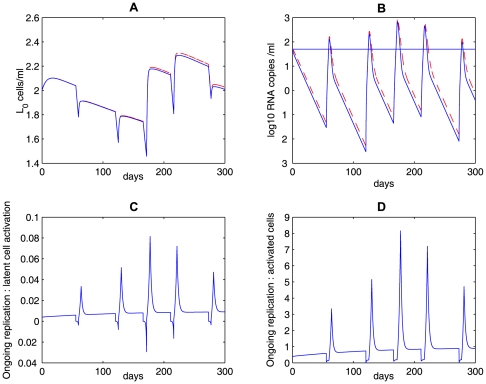
Relative contributions of ongoing viral replication and latent cell activation. **A and B:** the effects of ongoing viral replication (influenced by the overall drug efficacy) on the latent reservoir and viral load in the model given by Eq. (6). Different drug efficacies are used: 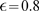 (red dashed line) and 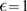 (blue solid line). Ongoing viral replication is only a minor contributor to the stability of the latent reservoir and low-level persistent viremia, as indicated by the minor effect of changing drug efficacy from 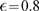 to 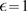. **C and D:** relative contributions of ongoing viral replication (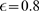 was fixed) and latent cell activation to the latent reservoir and viral persistence. **C:** the ratio of 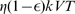 to 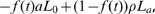, and **D:** the ratio of 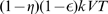 to 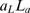. We chose 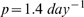. The other parameter values used are listed in Table 1.

The decay of the latent reservoir, the amplitude of viral blips, and the viral load below the limit of detection are not largely influenced by the effectiveness of the treatment as long as the overall drug efficacy is beyond a threshold value, 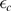, the critical drug efficacy. For this model we could not obtain a closed-form solution for 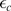 but it is numerically similar to that defined in (3). In Figures 7**A** and 7**B**, we explore the effects of HAART potency on the latent reservoir and low-level viremia by using different drug efficacies: 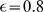 (red dashed line) and 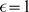 (blue solid line). Although 100% effectiveness may not be clinically feasible, we use an extreme case to illustrate the effect of latent cell activation. We observe that for the lower drug efficacy, the latent reservoir and the viral load are both at slightly higher levels. However, the difference is minuscule. This shows that both the stability of the latent reservoir and low-level persistent viral loads are principally due to latently infected cell activation rather than ongoing active viral replication, provided that the drug efficacy is above a certain threshold value.

We further compare the relative contributions of ongoing viral replication and latent cell activation to the latent reservoir and viral persistence. In Figure 7**C**, we plot the ratio of 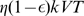 to 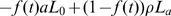, which represent the contributions to the latent cell pool coming from ongoing viral replication and the net effect of latent cell activation and return to latency, respectively. We find that the ratio is very small, indicating that the contribution of ongoing viral replication to the latent reservoir size is very small. In Figure 7**D**, we plot the ratio of 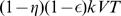 to 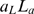, which represent the contributions to the viral load by *de novo* viral infection and the transition from activated latently infected cells into productively infected cells, respectively. The ratio is less than 1 except a few “blips” where latently infected cell activations occur. Thus, in the absence of activation, *de novo* viral infection is a minor factor contributing to the viral load, whereas viral blips are mainly due to *de novo* viral infection. However, we notice that the virus causing *de novo* viral infection is mainly released from latent cell activation (Figure 7**C**). Therefore, viral persistence and the stability of the latent reservoir arise primarily from occasional activation of latently infected cells upon antigen encounter. Residual active viral replication during HAART is only a minor factor.

**Figure 8:**
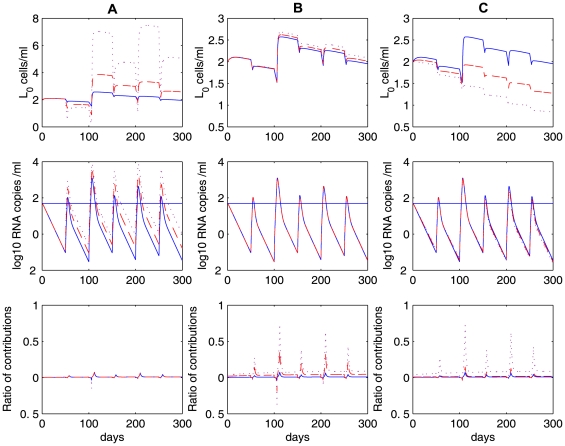
Sensitivity tests on several parameters when studying the relative contributions using model (6). The upper panels: the latent reservoir size; the middle panels: viral load; and the lower panels: the ratio of the relative contributions, i.e., the ratio of 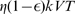 to 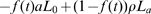. In column **A**, we use different activation rates: 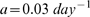 (blue solid), 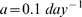 (red dashed), and 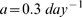 (purple dotted). There is no change in the ratio of relative contributions. In column **B**, we use different fractions of new infections that result in latency: 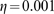 (blue solid), 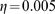 (red dashed), and 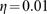 (purple dotted). In column **C**, we use different reversion rates to latency: 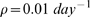 (blue solid), 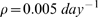 (red dashed), and 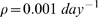 (purple dotted). The other parameter values used are the same as those in Figure 7.

We have also performed sensitivity tests on several parameters when studying the relative contributions. The ratio of 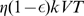 to 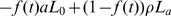 increases when we increase the activation rate of latently infected cells, 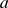, or the fraction of infections that result in latency, 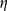, or decrease the reversion rate to latency, 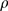. In Figure 8, we examined the effects of different parameter values of 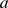, 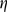 and 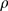 on the ratio of relative contributions. As the activation rate 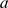 increases, more latently infected cells are activated, leading to more substantial replenishment of the latent reservoir and higher amplitudes of viral blips. However, even when 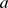 has a 10-fold increase (notice that in this case the transient viral load can reach above 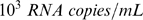, which is normally not regarded as a viral blip), we observe that the ratio of the relative contributions remains almost the same (Figure 8**A**). When the fraction of infections that lead to latency 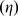 increases or the reversion rate to latency 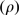 decreases, we observe similar effects on the viral load and the ratio of contributions (Figures 8**B** and 8**C**). Viral load does not change much. The ratio is far less than 1 (except when viral blips occur), supporting the conclusion that the latent reservoir persistence is mainly maintained by latently infected cell activation rather than ongoing viral replication. Notice that with a very large 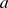 or 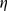 or a very small 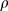 we do not generate viral blips with realistic amplitude (Figure 8**A**) or a slow decay of the latent reservoir (Figures 8**B** and 8**C**).

### Homeostatic proliferation of latently infected cells: another possible mechanism for the latent reservoir persistence

As shown in previous sections, occasional activation of latently infected cells upon antigen encounter can transiently produce a large number of activated cells, a small part of which can revert to the latent state and hence replenish the latent reservoir. In fact, several other sources might also reseed the latent cell pool and contribute to a stable latent reservoir: (1) homeostatic proliferation of 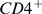 memory T cells regulated through combined effects of interleukin 7 (IL-7) and T cell receptor (TCR) signaling [57]; (2) “bystander” proliferation of latently infected cells induced by interferons or other cytokines released during the course of immune responses that do not cause the transition from the latent to active infection [30]; (3) latently infected cells generated during thymopoiesis (in which immature hematopoietic precursor cells mature after a series of replication, differentiation and selection steps) suggested by the SCID-hu (Thy/Liv) mouse model [58]; (4) latently infected cells transported from drug sanctuary sites or cells latently infected by virus released from drug sanctuary sites. A recent study by Chomont et al. [39] provides the first evidence supporting that the latent reservoir size and persistence can be maintained by homeostatic proliferation of latently infected cells. The proliferation of cells with provirus was also observed in another study [59]. Motivated by these mechanisms of reservoir replenishment, we include a logistic term that represents homeostatic proliferation of latently infected cells in the basic model (see Eq. (7) in Methods).

**Figure 9:**
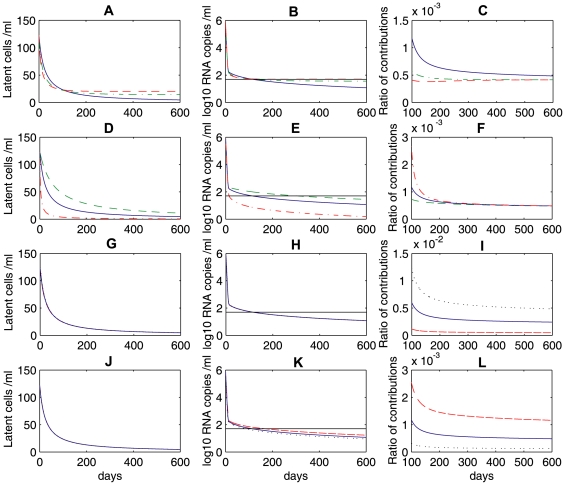
Numerical simulations of the homeostasis model (Eq. (7)) and sensitivity tests of several parameters. The system is at steady state and at 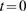 drug is applied. **A, D, G** and **J**: the latent reservoir size; **B, E, H** and **K**: viral load; **C, F, I** and **L**: the ratio of 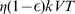 to 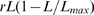, i.e., the relative contributions to the latent reservoir persistence from ongoing viral replication and latently infected cell proliferation. **A, B** and **C**: the carrying capacity of total latently infected cells is 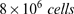. We use different proliferation rates: 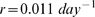 (blue solid), 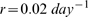 (green dash-dotted), and 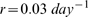 (red dashed). The black solid line represents the detection limit. **D, E** and **F**: 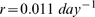 is fixed. Different carrying capacities of the total latently infected cells are used: 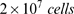 (green dashed), 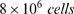 (blue solid), 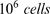 (red dash-dotted). **G, H** and **I**: we use different fractions of infections that result in latency: 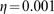 (red dashed), 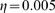 (blue solid), and 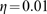 (black dotted). **J, K** and **L**: we use different drug efficacies: 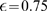 (red dashed), 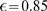 (blue solid), 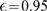 (black dotted). 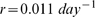 and the carrying capacity 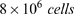 are fixed for the last two rows. The other parameter values used are listed in Table 1.

The homeostasis model can robustly describe the multiphasic viral decline following initiation of combination antiretroviral treatment, and maintain both low-level persistent viremia and the latent reservoir during therapy. Figure 9 shows the latent reservoir size, viral load and the ratio of relative contributions to the latent reservoir persistence of ongoing viral replication to latently infected cell proliferation with different parameter values. In the first row, we let the proliferation rate 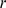 change but fix the total body carrying capacity of latently infected cells to be 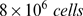 (i.e., 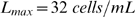). We observe that different latently infected cell proliferation rates yield different viral loads and different decay rates of the latent reservoir during the third phase. A larger 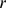 leads to higher levels of virus and latently infected cells. Consequently, a larger 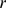 corresponds to a slower decay or a longer half-life of the viral load and the latent reservoir during the third phase (Figures 9**A** and 9**B**). In the second row, we show the changes in latently infected cells and viral loads with a fixed homeostatic proliferation rate, 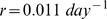. The total body carrying capacity of latently infected cells varies from 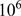 to 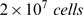 per patient (i.e., 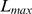 varies from 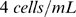 to 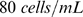). The larger carrying capacity, the higher levels of residual virus and latently infected cells (Figures 9**D** and 9**E**). It is interesting to observe that the time needed for the viral load to decline from the initial value 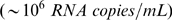 to below 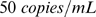 is short for a small carrying capacity. For example, when the total body carrying capacity is 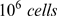 (red dash-dotted line, Figure 9**E**), it takes only about two weeks for the viral load to decline from 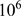 to 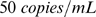. This is not typically observed in clinical trials. The first phase of viral decay causing 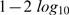 viral decline usually takes about 2 weeks and the viral load will not decrease to below the limit of detection until a few months after initiation of HAART [4]–[6]. This shortcoming can be overcome by incorporating a second infected cell population — long-lived infected cells [4]. In fact, Perelson et al. [4] proposed that the loss of long-lived infected cells, such as infected macrophages with the half-life of 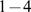 weeks, might be a major contributor to the second phase. In this section, for simplicity we do not include the long-lived population. We choose the total body carrying capacity to be 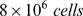 (blue solid line, Figure 9**E**) so that the viral load decreases to below 50 copies/mL after about three-month treatment.

We also plot the level of latently infected cells and viral load with different 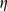, the fraction of new infections that result in latency (Figures 9**G** and 9**H**), and different 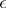, the drug efficacy (Figures 9**J** and 9**K**). As 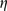 increases from 0.001 to 0.01, we do not find changes in the viral load or the latent reservoir size (Figures 9**G** and 9**H**). With the drug efficacy varying from 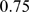 to 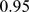 (ensuring that the viral load is suppressed to below the detection limit), we observe that the treatment potency has almost no effect on the latent reservoir (Figure 9**J**), although a higher drug efficacy always yields a lower viral load (Figure 9**K**). In our simulations, low levels of viremia persist in patients despite very effective drug treatment. This supports the idea that viral persistence under HAART is primarily due to the activation of latently infected cells.

We further examine the relative contributions to the latent reservoir persistence from ongoing viral replication and latently infected cell proliferation (i.e., the ratio of 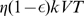 to 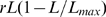) in the last column (Figures 9**C**, 9**F**, 9**I** and 9**L**). We plot the ratio beginning at 100 days after treatment because we are interested in the relative contributions to the latent reservoir persistence when viral load is suppressed to below the detection limit. The ratio is very small for a wide range of parameter values (Figures 9**C**, 9**F**, 9**I** and 9**L**). This suggests that latently infected cell proliferation rather than residual viral replication is major factor contributing to the latent reservoir persistence during effective treatment.

**Figure 10:**
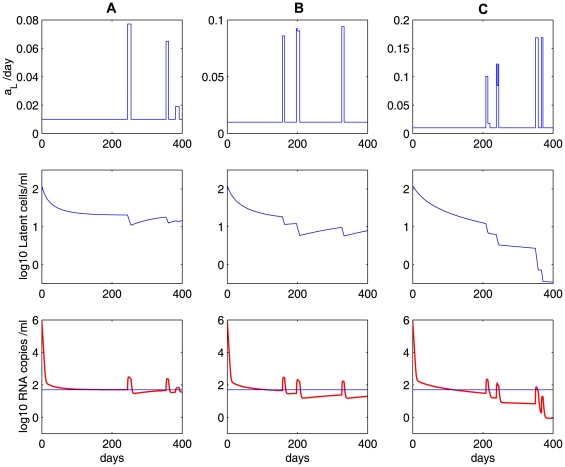
Simulations of the homeostasis model (Eq. (7)) with occasional increases of the transition rate 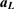. A Poisson process with an average waiting time of 2 months is used to model the random encounter between latently infected cells and antigens. We assume the total body carrying capacity of latently infected cells is 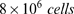. Column **A:** 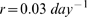; column **B:** 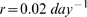; column **C:** 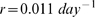. Different values of 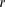 represent different potentials of latently infected cells to renew themselves, and thus lead to different decay rates of the latent reservoir. The other parameter values used are listed in Table 1.

The model with homeostatic proliferation of latently infected cells can also generate viral blips given intermittent bursts of activation of latently infected cells (i.e., increasing 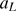 randomly) upon encounter with their specific antigens. Figure 10 delineates the transition rate 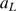, the time evolution of the latent reservoir and viral load with different proliferation rates: (**A**) 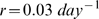; (**B**) 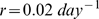; (**C**) 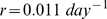. We use a Poisson process to model the encounter between latently infected cells and their relevant antigens. The average waiting time between two encounters is assumed to be two months. In the absence of high levels of specific antigen, the transition rate is assumed to be the base value, 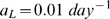. When the specific antigen is present, antigenic stimulation increases the transition rate by 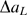. Some factors such as the antigen concentration and specificity may lead to different transition rates. Here we assume 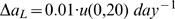, where 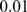 is the base value and 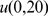 is a number chosen from a uniform distribution over the interval 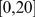. We further assume that the duration that each activation lasts obeys a normal distribution 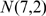. As the homeostatic proliferation rate 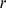 decreases from 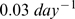 (Figure 10**A**) to 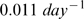 (Figure 10**C**), the decay rate of the latent reservoir increases. This is not surprising since with decreasing 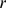 there is less self-renewal of latently infected cells. At a high enough proliferation rate, homeostasis of latently infected cells is able to maintain the latent reservoir size at a very stable level (Figure 10**A**).

The frequency of viral blips is also affected by the renewal potential since viral blips come directly from activation of latently infected cells. In the case of a weak renewal potential (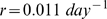, Figure 10**C**), a rapid decay of the latent reservoir may not generate a viral blip 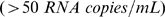 even if there is activation occurring. Thus, with a small 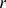, we expect a small number of viral blips over longer periods (similar to the case of a small 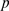 in Table 2). This also supports the observation that there is a strong inverse correlation between the decay of the latent reservoir and the number of intermittent viremic episodes observed per year [23].

## Discussion

Although several months of HAART is usually able to reduce the viral load in HIV-infected patients successfully to below the detection limit of standard assays, 50 RNA copies/mL, a low level of virus can still be detected in plasma by more sensitive assays [7],[8]. Despite many years of studies, there is still no single accepted explanation for the persistence of low-level viremia. It was initially thought that antiretroviral drugs would not be capable of completely suppressing residual viral replication, particularly in those sites that have poor drug penetration [60]. Changes in HIV-1 proviral sequences in peripheral blood mononuclear cells (PBMCs) [38], and several other lines of evidence (reviewed in [61]) also suggest that low-level ongoing replication exists in patients on HAART whose plasma HIV-1 RNA measurements are below the limit of detection.

However, even if HAART is potent enough to block all new infections of susceptible T cells, virus may still be released from a stable reservoir composed of latently infected 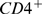 T cells. Studies of children with plasma virus levels below the detection limit showed that the existent virus lacked protease inhibitor resistance mutations despite the frequent use of the protease inhibitor nelfinavir, which has a low mutational barrier to resistance [62]. Protease sequences resembled those of virions from latently infected 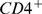 T cells, indicating that the low-level virus might originate from the latent reservoir. Amplification of plasma viral genomes in both children and adults showed that the low-level virus does not exhibit new, HAART-selected mutations and suggested that the viremia results primarily from archival, pre-HAART virus that comes from activation of latently infected cells [63]. Rebounding virus in patients after interruption of long-term potent treatment was also shown to originate from activation of latently infected cells [64],[65]. This further supports that the low-level persistent viremia during HAART comes from the latent reservoir since this continuously produced virus is most likely to rebound when treatment is stopped. Quantitative understanding of the factors and their contributions to persistent low-level viremia would provide valuable information that potentially could allow the design of more effective treatment strategies.

The mechanism for the stability of the latent reservoir in the setting of HAART remains controversial. The observation that intensification of antiretroviral therapy can accelerate the decay of the latent reservoir in some patients [66] suggests that residual viral replication may replenish the latent reservoir through *de novo* infection of susceptible cells on HAART [23],[56]. However, to what degree residual viral replication reseeds the latent reservoir in the setting of current HAART is still unclear. A recent study by Dinoso et al. [67] showed that treatment intensification could not reduce residual HIV-1 viremia in patients on HAART. Another explanation for the latent reservoir persistence stems from the intrinsic slow turnover of long-lived resting memory 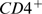 T cells [21],[22],[36],[68]. Recent evidence that some patients do not develop drug resistance despite long periods of HAART appears to support the hypothesis that the reservoir stability comes from the intrinsic stability of latently infected cells rather than ongoing viral replication [69]–[71]. Clearly understanding the nature of the reservoir stability is of significant importance since it is directly related to treatment strategies. If the latent reservoir is reseeded by low-level virus production due to the inability of antiretroviral therapy to completely suppress viral replication, then intensification of current regimens might help diminish the size of the reservoir. If the reservoir stability comes from the intrinsic stability of the latently infected cell population, then immune activation strategies or other means of flushing the reservoir have to be developed before virus eradication can be achieved.

Most well-suppressed patients with plasma HIV-1 RNA below the detection limit of 50 copies/mL demonstrate transient episodes of viremia above the limit (viral blips) [7],[23]. Because of non-intensive sampling, the characteristics of blips, such as the occurrence timing, frequency, duration and amplitude, are not well-known. The origin and the clinical significance of viral blips under seemingly effective antiretroviral treatment remain unclear. Viral blips may come from higher levels of virus production [72] due to transient reduced drug concentrations, or increased target cells secondary to opportunistic infection or vaccination [27], [33], [73]–[76]. They may also result from viral release from the latent reservoir because of heightened immune activation during vaccination or illness [27],[40],[63]. Nettles et al. [26] suggested viral blips could also be the result of laboratory error or statistical variation. Despite plasma HIV-1 RNA greater than the detection limit, viral blips have been reported not to be associated with virological failure [72], [77]–[79]. However, in some studies they have been associated with viral evolution [80], including the selection of drug resistant variants [81]–[83], an increased risk of clinical failure [55], and a slower decay of the latent reservoir [23]. Whether there exists the evolution of drug resistance during blips seems to depend on the amplitude of blips—the study by Easterbrook et al. [82] suggested that patients with transient viremia greater than 400 copies/mL are three times more likely to experience sustained viral rebound compared with those who maintain undetectable viral load.

In many mathematical models, the steady state viral load is very sensitive to small changes of drug efficacy and thus they cannot robustly describe the low viral load persistence during HAART [28]. It is also difficult to model the stability of the latent reservoir in the setting of potent treatment unless the latent cell death rate and the transition rate from the latent to productive state are extremely small [31] or balanced by bystander proliferation [30]. If viral blips also result from activation of latently infected cells, as suggested in [27],[40],[63], then this activation will accelerate the decay of latently infected cells and deplete the latent reservoir quickly, contrary to what was observed in clinical studies. In an attempt to examine whether intermittent viral blips can occur without seriously depleting the latent reservoir, we developed a new mathematical model that studies the latently infected cell response when cells encounter their relevant antigens.

Our model can robustly maintain the stability of the latent reservoir and meanwhile generate viral blips with reasonable duration and amplitude in infected individuals in the setting of HAART. We hypothesize that latently infected cells act similar to other memory cells and experience programmed proliferation and contraction upon antigenic stimulation by their recall antigens. During the response, a portion of activated latently infected cells transition into the productive class and generate viral blips. In the meanwhile, a small fraction of activated cells revert back to the resting state, providing a potential to replenish the latent reservoir. An interesting result is that this model can reconcile the divergent estimates of the decay rate of the latent reservoir in the literature. The half-life of the reservoir decay is largely determined by the frequency and duration of antigenic stimulation and by how many times the resultant activated latently infected cells proliferate during the latent cell response. In addition, we observe that assuming activated T cells remain at a low level after the rapid contraction phase (the biphasic decay model, i.e., Eq. (6)) can maintain a low level of viremia. This suggests that latently infected cell activation solely can maintain low-level viremic persistence and produce intermittent viral blips simultaneously, with the latent reservoir occasionally replenished. Model simulations show that the levels of persistent viremia and latently infected cells are not correlated with HAART potency, which suggests that low viral load persistence and the stability of the latent reservoir need not arise from ongoing active replication during HAART.

The conclusion that ongoing viral replication is a minor factor contributing to viral and the latent reservoir persistence is consistent with the results of recent studies [31],[32],[65],[84]. Using an assay capable of detecting HIV-1 RNA down to 1 copy/mL [8], Maldarelli et al. [84] suggested that more than 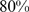 of patients on HAART had quantifiable viremias (with the median of 3.1 copies/mL) for at least two years after initiation of therapy, and that the level of persistent viremia was correlated with pretherapy viremia, or “set point”, but not with the specific treatment regimen (i.e., lopinavir/ritonavir versus nelfinavir as the protease inhibitor in HAART). These observations suggest that the persistent low-level viremia is derived from virus production by reservoirs infected prior to initiation of therapy, rather than ongoing viral replication during HAART. Bailey et al. [85] reported that in some patients a single, homogenous but distinct viral sequence (PPC) dominated the residual plasma virus but could not be readily found in the patient's resting 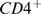 cells in peripheral blood. With the assumption that reservoir replenishment by ongoing viral replication in the presence of the PPC would eventually lead to incorporation of the PPC into the latent reservoir, and using a simple mathematical model to constrain the maximum rate of reservoir replenishment by viral replication [32], they suggest that ongoing viral replication during HAART is unlikely to be a major factor contributing to the stability of the latent reservoir. By characterizing rebounding virus during the structured treatment interruptions, Joos et al. [65] argue against persistence of ongoing low-level viral replication in patients under suppressive combination therapy.

Motivated by the observation that latently infected cells have the potential to renew themselves when stimulated by their previously encountered antigens, a much simpler phenomenological model with homeostatic proliferation of latently infected cells was proposed to study viral persistence and HIV-1 blips. The idea that the stability of the latent reservoir can be maintained by homeostatic proliferation of latently infected cells is also supported by a very recent experimental study [39]. Our model is able to simulate the multiphasic viral decay in patients after initiation of HAART. In this model, the homeostatic proliferation rate of latently infected cells is a key factor determining the half-life of the latent reservoir decay. A few factors, such as the concentration of antigen and its specificity, may affect the proliferation capacity of latently infected cells. In addition, the decay of the latent reservoir is inversely correlated with the frequency or amplitude of viral blips, as has been observed in the clinical study [23].

Considering that the latent reservoir consists of heterogeneous mixture of latently infected T-cell clones that respond differently to different antigens, our models can be generalized to account for the heterogeneity of latently infected cells. For example, we can extend the homeostasis model by including multiple subpopulations of latently infected cells, with each subpopulation having a different transition rate 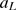 (see Eq. (7)). We expect that those subpopulations specific for frequently encountered antigens will be preferentially activated and removed from the reservoir, whereas those subpopulations that are specific for rarely encountered antigens may persist without activation or be activated slowly and dominate the latent reservoir. However, this prediction can be affected by the proliferation ability 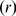 of each subpopulation. When we extend the model with programmed expansion and contraction by including multiple subpopulations of latently infected cells, the situation is a little different. Without assuming homeostatic proliferation of latently infected cells, depletion of one subpopulation does not imply other subpopulations will grow. The dynamics of each latently infected subpopulation depends on the proliferation ability of that subpopulation (i.e., the parameter 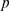 in Eq. (4)) and the reversion rate 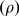 from the activated to latent state. Unfortunately, currently we do not have data on the heterogeneity of the latent reservoir that can be compared with these models.

Given that the latent reservoir has been identified as a major barrier to virus eradication with current combination therapy [86], elimination of this reservoir by novel therapeutic approaches is required before eradication can be achieved [87]. Our modeling results suggest that the stability of the latent reservoir is most likely due to the intrinsic stability of resting memory 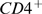 T cells and their occasional replenishment by antigenic stimulation. Thus, simply intensifying current therapy to further suppress ongoing active replication may not have much impact on the decay of the latent reservoir. Immune stimulation with activating agents has been proposed as a means to “flush” virus out of the latent reservoir. However, efforts to purge the latent reservoir with these agents have unfortunately shown only limited success. For example, although a combination of OKT3 and recombinant human IL-2 resulted in apparent T cell activation and proliferation [88], patients failed to achieve measurable purging of the cellular HIV reservoir. Moreover, side-effects were serious and antibodies against OKT3 developed rapidly in all patients. More importantly, if the activation of T cells in the latent reservoir also induces renewal of the latent pool as suggested by the models developed in this study, then activating agents could do more harm than good. Partial eradication of the latent reservoir will not be of significant benefit to infected individuals since theoretically a single infected cell has the potential to rekindle infection [89]. Therefore, a combination of activating agents with antiretroviral drugs can be useful only if they lead to a complete elimination of all the latent reservoirs.

Unfortunately, as viral levels are driven down, say below 1 RNA copy/mL, and latently infected cells become rare, it becomes impossible to follow the dynamics of these populations. Therefore, we must rely on mathematical models to make inferences about the end game in viral eradication. Here we have presented a set of models that agree with much of our knowledge about low-level viral persistence, the latent reservoir, and viral blips. Direct experimental tests of these models would involve the examination of the latently infected cell response when cells are activated by specific antigens. In addition, all of our models suggest that there is an inverse relationship between the decay of the latent reservoir and the frequency (or amplitude) of viral blips. Thus, more accurate and frequent data on the latent reservoir size, the number of viral blips and their amplitudes also can be used to test our models.
